# The Effects of Reduced Physical Activity on the Lipid Profile in Patients with High Cardiovascular Risk during COVID-19 Lockdown

**DOI:** 10.3390/ijerph18168858

**Published:** 2021-08-23

**Authors:** Marco Alfonso Perrone, Alessandro Feola, Massimo Pieri, Bruno Donatucci, Chiara Salimei, Mauro Lombardo, Andrea Perrone, Attilio Parisi

**Affiliations:** 1 Department of Systems Medicine, University of Roma Tor Vergata, 00133 Rome, Italy; chiara.salimei@gmail.com; 2 University Sports Centre, University of Rome Tor Vergata, 00133 Rome, Italy; brunodonatucci2@gmail.com; 3 Department of Experimental Medicine, University of Campania Luigi Vanvitelli, 80138 Naples, Italy; alessandro.feola@unicampania.it; 4 Department of Experimental Medicine, University of Rome Tor Vergata, 00133 Rome, Italy; massimo.pieri@uniroma2.it; 5 Department of Human Sciences and Promotion of the Quality of Life, San Raffaele Roma Open University, 00166 Rome, Italy; mauro.lombardo@uniroma5.it; 6 Department of Management Engineering, University of Rome Tor Vergata, 00133 Rome, Italy; andrea.perrone@live.it; 7 Department of Movement, Human and Health Science, University of Rome Foro Italico, 00135 Rome, Italy; attilio.parisi@uniroma4.it

**Keywords:** physical activity, COVID-19, LDL, cholesterol, cardiovascular risk

## Abstract

**Background:** The COVID-19 pandemic is a serious global health problem. In Italy, to limit the infections, the government ordered lockdown from March 2020. This measure, designed to contain the virus, led to serious limitations on the daily life of the individuals it affected, and in particular in the limitation of physical exercise. The aim of this study was to evaluate the effects of reduced physical activity on the lipid profile in patients with high cardiovascular risk. **Methods:** We enrolled 38 dyslipidemic patients, 56% male, with an age range of 44–62 years, considered to be at high cardiovascular risk. All patients were prescribed statin drug therapy (atorvastatin 40 mg) and a vigorous physical activity program four times a week, 1 h per session. In addition, a personalized Mediterranean diet was prescribed to all the patients. Total cholesterol, LDL, HDL and triglycerides were measured in patients at T0 before lockdown and at T1 during lockdown. **Results:** Data showed a significant increase (*p* < 0.01) in total cholesterol (+6,8%) and LDL (+15,8%). Furthermore, the analysis of the data revealed a reduction in HDL (−3%) and an increase in triglycerides (+3,2%), although both were not significant (*p* > 0.05). **Conclusions**: Our study showed that the reduction in physical activity during lockdown led to an increase in LDL levels, and therefore, in the risk of ischemic heart disease in dyslipidemic patients with high cardiovascular risk.

## 1. Introduction

Coronavirus disease 2019 (COVID-19) is a pandemic caused by severe acute respiratory syndrome coronavirus 2 (SARS-CoV-2), which has led to over 430.000 deaths worldwide today [1]. In 15% of infected patients, the clinical course of this pathology is complicated by the onset of a serious form of pneumonia, which can progress towards acute respiratory distress syndrome (ARDS) and or multi-organ failure (MOF) and death [2]. Italy was one of the countries most affected by COVID-19, with over 240.000 infections and 35.000 deaths [3]. In order to contrast the virus diffusion, the Italian government ordered the lockdown for the Italian population from 7 March 2020 [3]. This measure, designed to contain the virus, led to hard limitations on the daily life of the individuals it affected, and in particular led to the limitation of physical exercise [4]. This strategy was necessary for infection control but has potential behavioral and clinical repercussions.

Similar political decisions have been made in many countries [5].

Home isolation is likely to result in a profound decrease in physical activity levels, increase in sedentary behavior [4] and other adverse effects such as post-traumatic stress or symptoms of depression [5]. It is well established that regular physical activity is part of a healthy lifestyle [6,7]. Indeed, a plethora of experimental and clinical studies have definitively demonstrated that regular physical activity is associated with reduced cardiovascular risk and mortality rate for cardiac disease [6,7,8]. In particular, large, population-based, prospective observational studies showed that maintaining or improving fitness is associated with lower cardiovascular disease risk, whereas low cardiorespiratory fitness is associated with increased risk for myocardial dysfunction and of cardiac mortality rate [6,7,8]. Furthermore, it has been shown that there is a strong relationship between cardiorespiratory fitness and mortality from COVID-19 infection [9]. Regular physical activity has a positive impact on major cardiovascular risk factors, including dyslipidemia [10,11,12]. In addition to physical activity, proper nutrition plays a fundamental role in a healthy lifestyle and in the prevention of cardiovascular diseases [13,14]. In particular, the Mediterranean diet has been recognized as an effective tool for reducing cardiometabolic risk and cardiovascular events [15]. The Mediterranean diet uses olive oil as the predominant oil, is rich in fruits, vegetables, legumes, includes fish and is limited in red and processed meats and sweets. Among many benefits, the Mediterranean diet has been shown to reduce LDL-cholesterol, which represents one of the main factors in the formation of the atherosclerotic plaque [16]. An increased level of LDL-cholesterol is linked to an increase in the formation and progression of atherosclerotic plaque, and LDL-cholesterol levels are correlated with mortality in patients with ischemic heart disease [17,18]. After a 3-month follow-up period in a PREDIMED trial, the Mediterranean diet was reported to improve lipid profile [19]. The purpose of this study was to assess the effects of physical (in)activity and proper nutrition on the lipid profile in patients with a high cardiovascular risk during the lockdown due to the COVID-19 pandemic. The hypothesis tested was that the Mediterranean diet could offset, at least in the short term, the lack of regular physical activity on the lipid profile. This hypothesis is particularly alluring within the framework of lockdown periods of unpredictable duration.

## 2. Methods

The study enrolled 38 dyslipidemic patients, 56% male, with an age range of 44–62 years, at high cardiovascular risk, according to the recent guidelines for the management of dyslipidemia of the European Society of Cardiology (ESC) [20]. The clinical characteristics and risk factors of the patients are shown in Table 1. Patients were followed through a multidisciplinary therapeutic protocol in the Department of Cardiology and Sports Medicine and in the Department of Clinical Nutrition of the University of Rome Tor Vergata. All patients were prescribed statin therapy (atorvastatin 40 mg) and were under a physical activity program 4 times a week, 1 h per session. In addition, a personalized Mediterranean diet was already in place before the lockdown, as a part of a clinical prevention program. We evaluated total cholesterol, LDL-cholesterol, HDL-cholesterol and triglycerides in the last visit before lockdown (T0) and during lockdown (T1). Due to the various limitations imposed by lockdown, only 14 of the pilot’s patients, who already had scheduled visits to the hospital as a part of their periodic, clinical follow up, were able to repeat the venous sampling and measurement of the lipid profile biomarkers 36–42 days after T0. All patients were in lockdown during the study period. At T0 and T1, immediately after venipuncture, the samples were transported to the clinical biochemistry laboratory of the University of Rome Tor Vergata, centrifuged and immediately analyzed. All biomarkers were evaluated with immunoassays using the Architect Abbott platform (Abbott Diagnostics, Longford, Ireland). The study was approved by the local ethics committee (ID number 44.20) and all patients signed an informed consent. The study was conducted in accordance with the Helsinki declaration.

### Statistical Analysis

The normality of all the data were determined by using the Shapiro–Wilk normality test. All measurements were analyzed using a paired *t*-test to compare before vs. during lockdown, and a *p* value of less than 0.05 was considered statistically significant. The statistical analysis and data graphing were performed using a commercialized statistical software package Medcalc (Version 18.2.1, Ostend, Belgium). Reported data are expressed as mean ± SE and the percent changes (Δ% between T0 and T1) in the measured parameters were calculated using the formula = [(post-value/pre-value × 100) − 100] %.

## 3. Results

**Figure 1:**
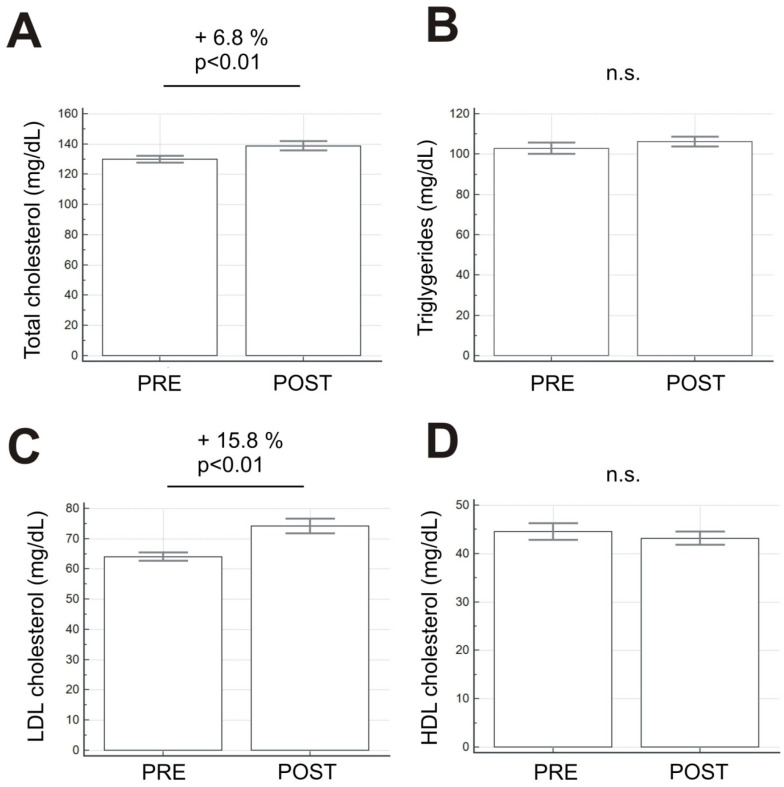
Changes in levels of total cholesterol (**A**), triglycerides (**B**), LDL (**C**) and HDL (**D**) cholesterol at T0 and T1. All values are expressed as mean ± S.E.M. (n.s. = not significant).

At T0, all patients had an optimal lipid profile, and the LDL levels, in particular, were <70 mg/dL, a therapeutic reference value for patients with high cardiovascular risk in accordance with the ESC guidelines [20]. During the lockdown, all 14 patients confirmed their full adherence to statin therapy and that they respected the indications of the prescribed personalized diet that was reported on a weekly, self-reported diary. All the patients reported that they had interrupted the prescribed physical activity program. All patients were in lockdown for six weeks. At T1, we observed a significant increase (*p* < 0.01) in total (+6,8%) and LDL-cholesterol (+15,8%). There was also a reduction in HDL-cholesterol (−3%) and an increase in triglycerides (+3,2%), although these changes were not significant (*p* > 0.05) (Figure 1). Of the 14 patients who were all in perfect therapeutic range at T0, only 4 (28%) had LDL <70 mg/dL at T1.

## 4. Discussion

The main finding of this study is that contrary to our original hypothesis, the Mediterranean diet alone is not capable of controlling and maintaining a healthy lipid profile in individuals at high risk for CV diseases interrupting their regular physical activity programs. The aim of this pilot study was to evaluate the effects of interrupting physical activity while consuming a personalized Mediterranean diet on the lipid profile in patients at high cardiovascular risk during the COVID-19 lockdown in Italy. The data showed that before lockdown, thanks to a multidisciplinary approach including physical activity and diet, in addition to statins, all the patients had LDL-c levels of less than the recommended value of 70 mg/dL. The LDL-c value is a fundamental parameter in patients with high cardiovascular risk, as underlined in the recent ESC guidelines [20], where LDL levels <70 mg/dL in these patients were considered as a target value for the prevention of ischemic heart disease. Several studies have shown that a reduction in LDL-c to this level decreases the risk of ischemic heart disease, the percentage of recurrence of myocardial infarction after the first event and, in general, cardiovascular mortality [21,22,23]. However, reaching LDL levels < 70 mg/dL is not always possible with drug therapy alone, but it is often achievable thanks to a multidisciplinary approach with the improvement of lifestyle through physical activity and a personalized diet [24]. In our study, during the lockdown, all patients still continued the prescribed Mediterranean diet, and statin therapy as well, but had to interrupt their regular physical activity. The lack of this component of our multidisciplinary prevention/therapy approach resulted in a worsening of the previously successful lipid profile. Hence, a single lifestyle change, such as the assumption of a personalized Mediterranean diet, appears insufficient in controlling lipid profile, one of the main CV risk factors. It is known that regular physical activity, mainly of aerobic type, induces favorable changes in lipid metabolism. However, just two weeks of reduced physical activity are sufficient to provide a decrease in cardiorespiratory fitness and multi-organ insulin sensitivity [25]. The reduction in physical activity was caused by the closure of gyms, swimming pools and sports fields. However, it should not be forgotten that due to the lockdown, many people have reduced time away from home due to smart working, the closure of non-essential work activities and the limitation of outdoor physical activity during phase 1 of the lockdown. In fact, Fitbit, Inc., an American company that develops wearable devices that track an individual’s physical activity level, recently shared physical activity data from 30 million users that shows a marked reduction in average step counts in almost all countries during the week ending 22 March 2020, as compared with the same period last year [4]. The reduction in daily steps reduces myofibrillar protein synthesis rates, upregulates muscle atrophy and disrupts lipid metabolism, with a more atherogenic profile in male adults [26]. High volumes of sedentary behavior are detrimental to one’s health, even in the presence of regular aerobic and/or resistance training. Sedentary behavior is defined as “any waking behavior characterized by an energy expenditure <1.5 METs, while in a sitting or reclining posture”, and it is important to counteract it with simple maneuvers such as getting up for short walks every 30 min. A sedentary lifestyle causes important alterations in metabolism, such as: reduced glucose tolerance, insulin resistance, reduced lipid clearance, visceral lipid deposition, reduced circulating levels of HDL and systemic inflammation. In particular, fat accumulation in liver likely stimulates de novo lipogenesis and an increased synthesis of atherogenic lipid particles. It is important that doctors advise their patients to spend as little time as possible sitting and lying down and to engage in low-intensity physical activities. Even short intermittent bouts of walking during the day reduce glucose and insulin concentrations in the postprandial period, mostly with more potent effects observed in adults with overweight to obesity and diabetes mellitus [27].

Sedentary behaviors can cause obesity, and the latter is a major risk factor for COVID-19 mortality [28]. Our study showed that stopping physical activity led to a significant increase (+15,8%) in LDL, as well as an increase in total cholesterol. Already, Sung et al. [29] showed in healthy athletes that an interruption of one month of training led to an increase of 9,3% of LDL. A change in the lipid profile in patients with high cardiovascular risk can even put their lives at risk by increasing the risk of ischemic heart disease [21]. Therefore, the prescription of physical activity must be considered as a real therapeutic approach to cardiovascular diseases, and perhaps patients should be incentivized not to interrupt physical activity by providing them with programs that can also be applied at home. In fact, many studies have demonstrated the feasibility, safety and efficacy of different models of home-based exercise programs [4,30,31]. To avoid premature deaths related to physical inactivity, health care professionals and public health agencies should act together in promoting physical activity during quarantine. Physical activity improves immune function via mobilizing lymphocytes and releasing cytokines such as IL-6, IL-7 and IL-15. Individuals with good cardiorespiratory fitness are less vulnerable to viruses, and therefore, maintaining regular movement is pivotal during lockdowns [5]. Caution should be exercised when prescribing home-based training for high-risk patients. For those, closer remote supervision using telecommunication might be necessary [4,32]. The COVID-19 pandemic has driven the rapid expansion of telemedicine use for urgent and non-urgent care visits, and it is also possible to monitor workouts and give live feedback [33].

Technological advancements over recent years have boosted the emergence of a multitude of tools, such as physical activity trackers and applications for smart watches and phones, that can help to monitor physical activity interventions [4]. This program is only viable through close collaboration between a multidisciplinary team (cardiologists, sports doctors, physiotherapists and exercise specialists) and patients. In fact, physical exercise prescription protocols and continuous monitoring are necessary. The intensity of the work must vary according to the physical condition and age of the individual. In planning and carrying out physical exercise sessions, it will therefore be important to follow certain indications in terms of the intensity, frequency, volume and mode of exercise. In the initial phase, it may be useful to alternate training days with rest days or to increase the volume of daily physical activity practiced over time. For aerobic activities, the guidelines recommend moderate intensity activities for most days of the week, with some of the individual sessions performed at a higher intensity. The ideal choice should therefore be to carry out moderate intensity activities, with a heart rate around 60–70% of the maximum heart rate. If heart rate cannot be monitored during exercise, the perceived level of fatigue should be considered, trying to manage exercise intensity between mild and moderately intense [34].

In patients with dyslipidemia and previous COVID-19 infection, exercise training can be an important part of cardiac rehabilitation, capable of inducing significant changes in the cardiovascular system and functional in the recovery of endothelial dysfunction and for the containment of thromboembolic complications [35]. Therefore, although several studies have shown that home programs involving low or moderate to vigorous intensity exercises have been shown to be safe and effective for patients with stable cardiovascular disease, care should be taken when prescribing home training for patients at high risk [36,37,38]. It is certainly safer for these patients to engage in closer remote supervision using telecommunications, both of the training program and especially of the vital parameters and of the clinical and cardiovascular conditions [4,34].

With personalized physical activity programs and home clinical monitoring it may be possible to restore multidisciplinary therapeutic programs (drugs, physical activity and diet) for patients with high cardiovascular risk in peculiar situations such as the COVID-19 lockdown [36]. Compatibly with the restrictions imposed by governments, an alternative to training at home is to train outdoors (for example, in parks). It has in fact been shown that the risk of contracting COVID-19 is low when training in open spaces and when respecting social distancing [39].

Indeed, there is increasing interest in the health benefits of parks and other urban green spaces, because many studies have demonstrated that exposure to green spaces in urban environments is associated with improvements in physical and mental health [40].

Maintaining LDL levels <70 mg/dL for these patients is a key goal for the prevention of ischemic heart disease events [18]. On the other hand, in patients at high cardiovascular risk who perform regular physical activity, it has been shown that a change in the frequency of physical activity can have important repercussions on the risk of ischemic heart disease. Kim et al. [41] demonstrated that changes in the frequency of moderate to vigorous physical activity (MVPA) can result in different cardio-metabolic risk profiles. In particular, a reduction in the frequency of MVPA increases the cardiovascular risk by 27%, while an increase in the frequency of MVPA reduces the risk of cardiovascular events (coronary heart disease and stroke) by 9%.

### Study Limitations

The main limitation of the present investigation is the small sample size, furtherly penalized by lockdown. As such, the study should be regarded as a “proof-of-concept” study. For the same reason, we cannot comment on possible gender-related differences. Finally, we also cannot comment on clinical outcomes. However, clinical outcome was not the focus of the study.

## 5. Conclusions

Our study showed that the reduction in physical activity during lockdown due to COVID-19 can worsen the lipid profile, and therefore the risk of ischemic heart disease, in dyslipidemic patients with high cardiovascular risk. While waiting for vaccinations to contain the COVID-19 epidemic, it is important that people with cardiovascular risk factors exercise safely (by training at home or outdoors, respecting social distancing) both to prevent the progress of cardiovascular pathologies and because the sedentary lifestyle and its consequences at the same time worsen the clinical course of a possible COVID-19 infection. The impact of physical activity as a therapeutic aid for these patients should be supported by a personalized home exercise program to cope with the inability to go to dedicated sports facilities. Considering the increases in infection in recent weeks and the risk of a new lockdown, surely a multidisciplinary project of home care through hi-tech and telecommunications devices could be of fundamental importance for these patients. More studies will be needed to verify the feasibility and effectiveness.

## Figures and Tables

**Table 1:** The table shows the clinical characteristics and cardiovascular risk factors (%) of the 14 patients studied.

Age (years)	54,6 ± 5,1
Weight (kg)	74,9 ± 6,3
Height (cm)	166,9 ± 5,0
BMI (kg/m^2^)	26,9 ± 2,1
Dyslipidemia (%)	100
Hypertension (%)	57,1
Diabetes (%)	14,3
Cigarette smoke (%)	28,6
Sex (M:F)	9:5
